# ERP Effects of Malicious Envy on Schadenfreude in Gain and Loss Frames

**DOI:** 10.3389/fnhum.2021.663055

**Published:** 2021-08-12

**Authors:** Huiyan Lin, Jiafeng Liang

**Affiliations:** ^1^Institute of Applied Psychology, School of Public Administration, Guangdong University of Finance, Guangzhou, China; ^2^Laboratory for Behavioral and Regional Finance, Guangdong University of Finance, Guangzhou, China; ^3^School of Education, Guangdong University of Education, Guangzhou, China

**Keywords:** malicious envy, schadenfreude, gain, loss, ERPs

## Abstract

Previous behavioral and neural studies have shown the effects of malicious envy on schadenfreude. However, it is unclear whether these effects are modulated by contextual frames (e.g., gain and loss frames). Thus, the present study aimed to investigate whether behavioral and event-related potential (ERP) effects of malicious envy on schadenfreude were different in gain and loss frames. To address this issue, the participants in the present study believed they were playing a monetary game with several other players. In the malicious envy condition, the participants won less money than the player in the gain frame and lost more money in the loss frame; in the control condition, both participants and the player gained little money in the gain frame and lost much in the loss frame. Subsequently, the participants were informed that the player encountered a misfortune, i.e., gained little in the gain frame and lost much in the loss frame. Results showed that malicious envy increased feelings of schadenfreude and ERP responses when the player encountered a misfortune. Moreover, increased ERP responses by malicious envy occurred at the feedback-related negativity (FRN), and early late positive potential (LPP) time ranges in the gain frame but at the late LPP time range in the loss frame. The findings might suggest that malicious envy affects schadenfreude and corresponding neural activity, whereas the neural effects occur at comparatively early time ranges in the gain frame but at a later time range in the loss frame.

## Introduction

Envy is a social-comparison-based emotion that arises when we compare ourselves unfavorably with superior quality, achievement, or possessions of another (Parrott and Smith, 1993). Envy is thought to be one of the most potent causes of unhappiness (Russell, 1930) and has widespread effects on behaviors and other emotions of individuals, such as schadenfreude (i.e., malicious joy). Theories on envy [e.g., the malicious envy theory (Miceli and Castelfranchi, 2007; Smith and Kim, 2007), the dual envy theory (Van de Ven et al., 2009; Falcon, 2015), and the pain-driven dual envy theory (Lange et al., 2018)] have suggested that envious persons will feel schadenfreude when a misfortune befalls enviable persons due to the achievement of the motivation goal of envy (Van de Ven et al., 2015). In the present study, we further investigated in which circumstance envy influences schadenfreude and related neural mechanisms.

Previous empirical studies have utilized a scenario task to investigate the effect of envy on schadenfreude (e.g., Feather and Sherman, 2002; Feather and Nairn, 2005; Van Dijk et al., 2006; Takahashi et al., 2009; Feather et al., 2013; Van de Ven et al., 2015; Baez et al., 2016, 2018; Santamaría-García et al., 2017). In this task, individuals are told virtual stories about a protagonist (e.g., Van Dijk et al., 2006; Takahashi et al., 2009; Feather et al., 2013; Baez et al., 2016, 2018; Santamaría-García et al., 2017), or they are asked to imagine a virtual person or to describe a real person in everyday life (e.g., Feather and Sherman, 2002; Feather and Nairn, 2005; Van de Ven et al., 2015). To elicit envy, the protagonist or imagined/described person is superior to the individuals in a specific domain (e.g., study). Subsequently, individuals are told that the abovementioned superior protagonist encounters an unfortunate event (e.g., he/she fails in an important examination), or they are asked to imagine that the person whom they just imagined or described has encountered such an event. The feelings of pleasure (i.e., schadenfreude) and/or neural responses are assessed for this event. Notably, imagining or describing enviable persons and unfortunate events is not suitable for neural studies. Neural studies often require multiple trials to reduce artifacts. If such a task is used, participants have to imagine/describe a large number of enviable persons or the same person for multiple times. However, imagining/describing a large number of persons is difficult for the participants, and multiple repetitions will reduce the strength of the effect.

Using the abovementioned task, several behavioral studies have shown that envy increases feelings of schadenfreude (e.g., Van Dijk et al., 2006; Takahashi et al., 2009; Cikara and Fiske, 2013; Feather et al., 2013; Baez et al., 2016, 2018; Santamaría-García et al., 2017). However, some other studies have revealed that feelings of schadenfreude did not result from envy (Feather and Sherman, 2002; Hareli and Weiner, 2002; Feather and Nairn, 2005; Leach and Spears, 2008; Brambilla and Riva, 2017). Possible reasons for the discrepant findings might be associated with the category of envy and the social identity of enviable persons. Envy is thought to be categorized into malicious envy and benign envy (Smith and Kim, 2007; Van de Ven et al., 2011a,b; Van de Ven, 2017). Malicious envy tends to lead to resentment and a desire for revenge against others, while benign envy helps enhance self-elevation motivation. Further studies have shown that feelings of schadenfreude are increased by malicious envy rather than benign envy (Van de Ven et al., 2015; Lange et al., 2018). In terms of social identity, it was found that the effect of envy on feelings of schadenfreude was significant only when enviable persons were competitive out-group members (Cikara and Fiske, 2013) and served as a relevant social comparison (e.g., individuals and enviable persons had the same sex; Van Dijk et al., 2006).

Regarding neural studies, functional magnetic resonance imaging (fMRI) study of Takahashi et al. (2009) observed that activation was increased in ventral striatum and prefrontal cortex (e.g., medial orbitofrontal cortex) when the participants were reading a story in which an enviable protagonist encountered an unfortunate event. Similarly, a brain injury study by Baez et al. (2016) reported that patients who had Huntington’s disease and their healthy relatives (i.e., descendants or siblings who did not present any symptoms of Huntington’s disease or other neuropsychiatric diseases) had a reduced effect of envy on the feeling of schadenfreude compared with healthy controls who were not relatives of the patients with Huntington’s disease. As individuals with manifest and pre-manifest Huntington’s disease are thought to have impairments in the ventral striatum, the findings in the study of Baez et al. (2016) might suggest that ventral striatum is associated with the effect of envy on schadenfreude. Another brain injury study by Santamaría-García et al. (2017) showed that, under the influence of envy, feelings of schadenfreude were stronger for patients with frontotemporal dementia (circumscribed degeneration associated with the prefrontostriatal network) than for healthy controls and even Alzheimer patients, suggesting the role of prefrontal cortex and ventral striatum on the effect of envy on schadenfreude. Taken together, the abovementioned studies might imply that the ventral striatum and prefrontal cortex are associated with the effect of envy on schadenfreude. Moreover, as these brain regions are relevant to reward processing, the abovementioned findings might also indicate that envy influences reward processing associated with schadenfreude.

In general, previous studies have suggested that (malicious) envy influences feelings of schadenfreude and neural responses associated with reward processing when enviable persons encounter a misfortune. It is notable that some studies, in particular neural studies, used a virtual social comparison scenario to elicit envy (e.g., a virtual story about a superior protagonist; Takahashi et al., 2009; Baez et al., 2016; Santamaría-García et al., 2017). Using such a virtual social comparison scenario might have reduced the ecological validity of the effects of malicious envy on schadenfreude. While several other studies used real social comparison scenarios (e.g., a scenario regarding an enviable person in real life; Feather and Sherman, 2002; Feather and Nairn, 2005; Van de Ven et al., 2015), such scenarios are not suitable for neural studies due to the abovementioned repetition issue. Thus, looking for real and repeatable social comparison scenarios might be important in investigating the effects of malicious envy on schadenfreude, particularly the neural effects. In fact, previous studies have proposed such scenarios to elicit malicious envy, even though the scenarios are not used to investigate the effect of malicious envy on schadenfreude (e.g., Shamay-Tsoory et al., 2009; Dvash et al., 2010; Steinbeis and Singer, 2013, 2014). For instance, in the study of Steinbeis and Singer (2014), the scenario involved a monetary game between participants and players. Malicious envy was elicited as the difference between the condition in which the participants lost some money and the player won the money and the condition in which both the participants and the players lost money. In this case, real social comparisons could be repeatedly elicited by manipulating relative monetary outcomes between the participants and the players.

More importantly, these abovementioned studies did not consider in which contextual frame envy and schadenfreude occurred. Specifically, envy can be elicited in either a gain frame (e.g., both enviable protagonists/persons, and the participants obtain a pleasant outcome; and the outcome of the enviable protagonists/persons is more pleasant) or a loss frame (e.g., both enviable protagonists/persons and the participants obtain an unpleasant outcome, and the outcome of the enviable protagonists/persons is less unpleasant). Similarly, schadenfreude could also occur in both gain and loss frames (e.g., the enviable protagonist/person obtains a less pleasant or a more unpleasant outcome than other outcomes). It has been suggested that individuals in the gain frame focus on not only their own outcome but also the outcome of other individuals, whereas those in the loss frame are more likely to focus on their own outcome (De Dreu et al., 1992, 1994; Poppe and Valkenberg, 2003). As the effect of envy on schadenfreude occurs in the context of social comparisons, individuals might pay more attention to the outcome of enviable protagonists/persons in the gain frame than in the loss frame, leading to different effects of envy on schadenfreude.

Taken together, the present study aimed to investigate whether a contextual frame influenced the behavioral and neural effects of malicious envy on schadenfreude by using a real social comparison task. To address this issue, the participants were asked to play a monetary game with several other players. The participants and the players gained some money in the gain frame and lost money in the loss frame. In the experimental (i.e., malicious envy) condition, the outcome was better for the players than for the participants (i.e., the players gained more money than the participants in the gain frame and lost less money in the loss frame); in the control condition, both the players and the participants obtained a bad outcome (i.e., gained little money in the gain frame and lost much money in the loss frame). Subsequently, the players obtained a bad outcome. The participants were required to assess the feeling of schadenfreude for this bad outcome.

As mentioned above, different attentional allocations to outcomes of others in gain and loss frames might influence the effect of malicious envy on schadenfreude. This influence might take place in two ways: first, the effects of malicious envy on schadenfreude might be stronger in the gain frame than in the loss frame; and second, the effects might occur earlier in the gain frame than in the loss frame. Due to low temporal resolutions, however, the second hypothesis could not be understood by behavioral, fMRI, or brain injury techniques. To investigate both of these two ways simultaneously, techniques with high temporal resolutions [e.g., event-related potentials (ERPs)] should be used. Two ERP components [i.e., feedback-related negativity (FRN) and late positive potential (LPP)] might reflect the effect of malicious envy on schadenfreude. The FRN, which starts at approximately 200 ms with a stimulus onset and is distributed over anterior scalp sites, is thought to reflect expectancy violation. The response is more negative for unexpected outcomes than for expected outcomes (e.g., Bellebaum and Daum, 2008; Walsh and Anderson, 2012; Meadows et al., 2016). In a later time range, LPP (overlapping P300) develops for approximately 300 ms with the stimulus onset and sometimes lasts for a few seconds and is maximal over parietal scalp sites. This component is supposed to be relevant to outcome evaluations, with larger amplitudes for positive evaluations than for bad evaluations (e.g., Wu and Zhou, 2009; Walsh and Anderson, 2012; Meadows et al., 2016). Note that the LPP at earlier time ranges might overlap with the FRN and thus present an effect similar to FRN. More importantly, these two components have been found to reflect the activity of the ventral striatum and prefrontal cortex, the brain regions associated with the effect of malicious envy on schadenfreude (e.g., Carlson et al., 2011; Campanella et al., 2013; Pfabigan et al., 2014; Moore et al., 2019). Therefore, FRN and LPP might be used to investigate the modulation of a contextual frame on the effects of malicious envy on schadenfreude in different time ranges.

Based on previous studies, we predict that, in the present study, malicious envy would generally increase feelings of schadenfreude and ERP responses (e.g., FRN and LPP responses) when enviable persons encounter misfortune. Moreover, as mentioned above, previous studies have suggested that individuals are more sensitive to outcomes of others in the gain frame than in the loss frame (De Dreu et al., 1992, 1994; Poppe and Valkenberg, 2003). This processing pattern in the gain frame might allow the response of individuals to be faster and/or larger when attending to the misfortune of enviable persons. Accordingly, we predict that the behavioral and ERP effects of malicious envy on schadenfreude would be stronger in the gain frame than in the loss frame and/or that the ERP effects would take place at earlier time ranges (i.e., at FRN and even early LPP time ranges in the gain frame and at relatively late LPP time ranges in the loss frame).

## Materials and Methods

### Participants

Thirty-six undergraduate students were recruited from Guangdong University of Education. Two were excluded due to being given wrong instructions, and another two were excluded due to EEG artifacts. Therefore, the reported data were from 32 participants (ranging from 19 to 24 years old, *M* = 20.32, *SD* = 1.04; 20 females). All the participants were right-handed, as is determined by the Edinburgh Handedness Inventory (Oldfield, 1971). Previous studies, using factorial design, have revealed behavioral and neural effects of (malicious) envy on schadenfreude by using no more than 30 participants (e.g., Van Dijk et al., 2006; Takahashi et al., 2009; Baez et al., 2016, 2018; Santamaría-García et al., 2017). We also performed a pilot study with the same paradigm of the present study by another group of 30 participants (ranging from 18 to 22 years old, *M* = 19.79, *SD* = 0.85; 17 females), and the results showed a significant effect of malicious envy on feelings of schadenfreude. Thus, the sample size in the present study was sufficient. The participants reported normal or corrected-to-normal vision and no history of neurological illness. All the participants gave written informed consent in accordance with standard ethical guidelines as defined in the Declaration of Helsinki. The study was approved by the local ethics committee.

### Procedure

The participants sat in a comfortable chair in a quiet room approximately 100 ms directly in front of a 22-in computer monitor with a screen resolution of 640 × 480 pixels. Stimulus presentation and behavioral data collection were controlled by E-Prime 2.0 software (Psychology Software Tools, Inc., Sharpsburg, PA, United States). All stimuli were presented against a dark background.

Prior to the actual experiment, each participant was told that he/she would play a monetary game with three anonymous players. As the effect of malicious envy on schadenfreude is more evident when enviable persons are competitive out-group members (Cikara and Fiske, 2013) and serve as a relevant social comparison (e.g., the enviable persons were of the same sex as the participants; Van Dijk et al., 2006), the present study emphasized that the players were undergraduate students from other departments or schools in the university and that they were of the same sex as the participants themselves. The participants were informed that the players would play the game in other rooms, and the participants and players could not see one another. In fact, there were no players, and all the choices of the players in the experiment were predetermined by experimental randomization. To allow the participants to believe in the existence of the players, experimenters pretended to connect with other experimenters in the other rooms by mobile phones and talked about the players (e.g., the players have finished the exercise and have got ready). The participants were told that monetary gain or loss would be based on 50% of the general gain or loss across all trials, with the addition or subtraction of a basic compensation (e.g., 30 RMB), respectively [e.g., if the participants gained 10 RMB over all the trials in the game, then they would receive (30 + 10 × 50%) RMB. If they lost 10 RMB in the game, then they would receive (30−10 × 50%) RMB]. In fact, the general gain or loss was randomized by a computer and ranged from −9 RMB to +9 RMB.

**FIGURE 1:**
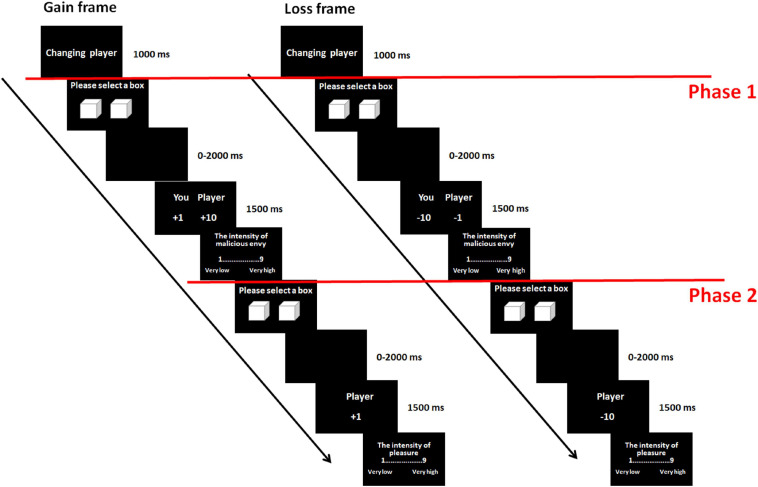
Experimental procedure in gain and loss frames (on **left** and **right** panels, respectively).

As is illustrated in Figure 1, the actual experiment consisted of gain and loss frames. These two frames were presented in different blocks, and the presentation sequence was counterbalanced across the participants. For both the gain and loss frames, each trial started with a label “changing player” for 1,000 ms. The label signified that the computers would select one of the three players in a randomized order for the next trial of the game. However, which person would be the player was unknown to the participants. Each trial consisted of two phases. The first phase was to elicit malicious-envy and nonmalicious-envy emotions, and the second phase was to assess feelings of schadenfreude. During Phase 1, the participants were presented with two white boxes, one to the left of the center and the other to the right. The participants were told that there was either 1 or 10 RMB in each box and that they would gain or lose that amount of money according to their selections. The participants were informed that this was a game of chance and that there was no relationship between the location of the box and the amount of money. The participants were told to choose one of the two boxes by pressing the “D” or “F” key for the left or right box, using the middle or index finger, respectively, of their left hands. There was no time limit for the response. Subsequently, a blank screen was shown in a time range between 0 and 2,000 ms (*M* = 1,000 ms). The participants were told that the blank screen would appear when their response was faster than the response of the player. This manipulation allowed the participants to believe they were playing with real persons. Then, the outcomes of the participant and the player were presented on the left and right sides, respectively, of the center for 1,500 ms. The number presented signified the amount of money gained or lost. On the left side of the number, there was a symbol “+” in the gain frame to indicate a monetary gain and a symbol “−” in the loss frame to indicate a monetary loss. The participants were then asked to rate how much malicious envy they felt toward the player on a nine-point scale (1 = very low, 9 = very high) by pressing the number on the number keypad of the keyboard, using the right hand. Given that previous studies showed that the effect of envy on schadenfreude was evident when envy was malicious rather than benign (Van de Ven et al., 2015; Lange et al., 2018), experimenters emphasized to the participants that the ratings referred to malicious envy rather than benign envy. The meanings of these two categories of envy and their differences were also explained to the participants (Smith and Kim, 2007; Van de Ven et al., 2011a,b; Van de Ven, 2017). Phase 2 started immediately after the envy assessment. This phase was similar to Phase 1, except for the outcome and the rating. The outcome presented after the box selection showed the outcome only for the other player but not for the participant. The rating reflected the intensity of pleasure that the participants felt upon seeing the outcome of the player [ranging from 1 (very low) to 9 (very high)]. Notably, to reduce social desirability issues, the participants in the present study were asked to rate the degree of pleasure, but not of schadenfreude, during the assessment of schadenfreude. This manipulation was consistent with previous studies (e.g., Takahashi et al., 2009; Steinbeis and Singer, 2013, 2014; Van de Ven et al., 2015; Baez et al., 2016; Santamaría-García et al., 2017). At the end of the experiment, the participants were asked whether they had participated in similar psychological experiments before and whether they actually believed in the existence of the other players. All the participants reported that they never had an experience with similar experiments and that they believed they had played with real persons.

**FIGURE 2:**
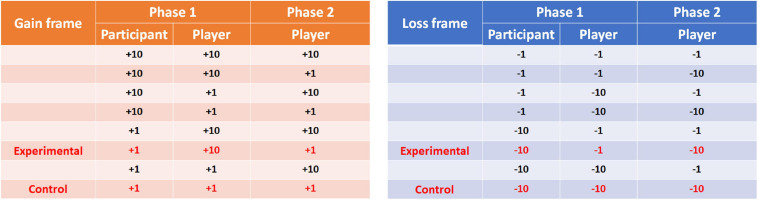
The outcomes between the participants and the players for the two phases of the game in gain and loss frames (on the **left** and **right** panels, respectively). The outcomes in red are those in the experimental and control conditions. Phase 1 elicited envy and non-envy emotions, and Phase 2 assessed the feelings of pleasure (schadenfreude).

The outcomes of the participants and players were, in fact, predetermined *via* experimental randomization. According to the outcomes for both the participants and players in Phase 1 and for the players in Phase 2, Figure 2 presents eight outcome combinations for the gain frame and eight for the loss frame. The outcome combinations in red were used in the experimental and control trials, and the other combinations were used in the filler trials. For the experimental condition, the outcomes for Phase 1 involved the participants gaining less money than the players in the gain frame (i.e., participants versus players = +1 versus +10) or losing more money in the loss frame (i.e., −10 versus −1). Steinbeis and Singer (2014) showed differential feelings of (malicious) envy between the experimental and control conditions when the outcome difference between the participants and other players was eight tokens in the experimental condition. Thus, in the present study, the outcome difference between the players and the participants in the relevant condition might be sufficient to elicit malicious envy. In the control condition, the outcomes involved both the participants and the players gaining a small amount of money (+1 versus +1) or losing a large amount of money (−10 versus −10). In both the experimental and control conditions, the player encountered comparative misfortune in Phase 2, i.e., gained a small amount of money (i.e., +1) in the gain frame and lost a large amount of money (i.e., −10) in the loss frame.

For each frame, there were 45 trials in the experimental condition and 45 trials in the control condition. Filler trials were presented 10–20 times each, for a total of 200 times (100 times/frame × two frames). Therefore, the actual experiment consisted of 380 trials (i.e., 45 trials/condition × four conditions + 200 trials). In each frame, there were five breaks. The duration of the break was controlled by the participants. Prior to the actual experiment for each frame, there were eight practice trials so the participants could familiarize themselves with the experimental procedure. The experiment (including practices) lasted approximately 2.5 h.

### Behavioral Recordings

Malicious envy and schadenfreude ratings were recorded for each trial. The ratings were averaged for all trials separately for each level of combination of emotion and frame.

### EEG Recordings and Preprocessing

Electroencephalograms (EEGs) were recorded, using two 32 BrainAmp amplifiers (Brain Products GmbH, Munich, Germany). Ag/AgCl electrodes were placed on the scalp by means of an EasyCap electrode system (EASYCAP GmbH, Herrsching-Breitbrunn, Germany) in the accordance with the 10–20 system. The electrode AFz was used as the ground electrode. The other channels were referenced to the electrode FCz online. The horizontal electrooculogram (EOG) was recorded from two electrodes at the outer canthi of both eyes, and the vertical EOG was recorded bipolarly from two electrodes above and below the right eye to monitor eye blinks and movements. The EEG was amplified, using a 0.016–100 Hz bandpass filter and a 50 Hz notch filter, and sampled at 1,000 Hz/channel. Electrode impedances were maintained below 10 kΩ.

Offline, EEG data were further processed, using BrainVision Analyzer 2.0 software (Brain Products GmbH, Munich, Germany). Raw data were re-referenced to the average of the left and right mastoids. Ocular movements were inspected and removed from the EEG signal, using the algorithm by Gratton et al. (1983). The continuous EEG was then segmented from −200 to 1,500 ms relative to the onset of the outcome of the player in Phase 2, with the first 200 ms epoch for baseline correction. Epochs containing artifacts exceeding 100 μV were excluded from averaging. Artifact-free trials were averaged for each channel and experimental condition. Averaged ERPs were then low-passed filtered at 30 Hz (24 db/oct, zero phase shift). Mean number of trials in the gain-experimental, gain-control, loss-experimental, and loss-control conditions was 41.75, 41.88, 41.46, and 42.22, respectively.

**FIGURE 3:**
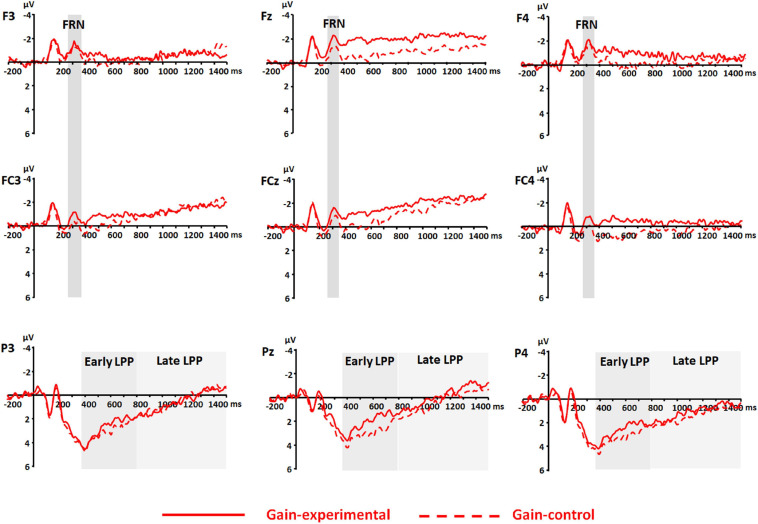
Mean ratings on malicious envy (on the **left** panel) and pleasure (schadenfreude, on the **right** panel) in each experimental condition. Error bars show 95% confidence intervals and are adjusted for within-subject designs. The significance level of the emotional effect is marked by the number of “*” symbols. “*” and “***” indicate *p* < 0.05 and 0.001, respectively. Note that the significant values refer to the main effect of emotion from the ANOVA.

**FIGURE 4:**
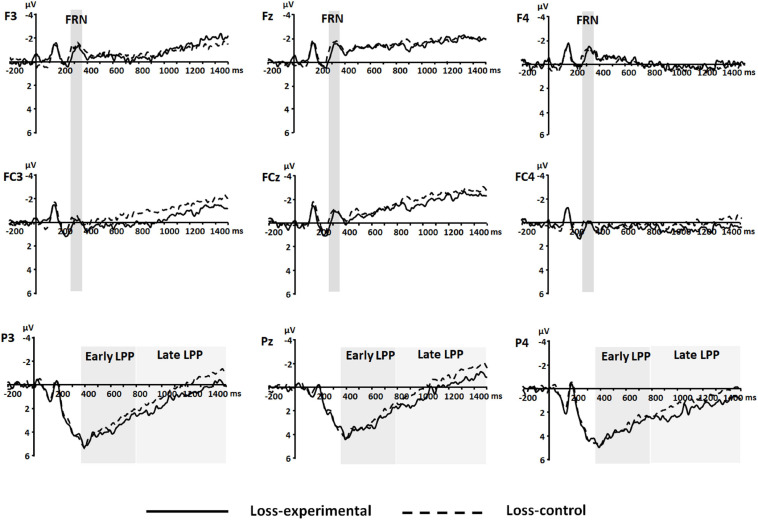
Event-related potential (ERP) waveforms at frontal (i.e., F3, Fz, and F4), frontocentral (i.e., FC3, FCz, and FC4), and parietal (i.e., P3, Pz, and P4) electrodes for the experimental and control conditions in the gain frame. Shaded areas represent time windows for feedback-related negativity (FRN) (264–364 ms) and early and late positive potential (LPP) (364–800 ms and 800–1,500 ms, respectively). The presented waveforms were obtained by averaging the waveforms across trials (*means* of trial numbers were 41.75 and 41.88 in the experimental and control conditions, respectively) and of 32 participants within the respective conditions.

**FIGURE 5:**
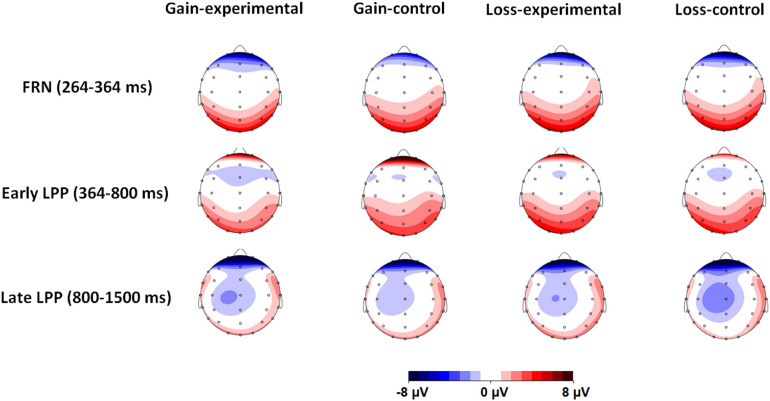
Event-related potential (ERP) waveforms at frontal (i.e., F3, Fz, and F4), frontocentral (i.e., FC3, FCz, and FC4), and parietal (i.e., P3, Pz, and P4) electrodes for the experimental and control conditions in the loss frame. Shaded areas represent time windows for feedback-related negativity (FRN) (264–364 ms) and early and late positive potential (LPP) (364–800 ms and 800–1,500 ms, respectively). The presented waveforms were obtained by averaging the waveforms across trials (*means* of trial numbers were 41.46 and 42.22 in the experimental and control conditions, respectively) and of 32 participants within the respective conditions.

Event-related potentials were qualified, using the mean amplitudes for FRN (264–364 ms), early LPP (364–800 ms), and late LPP (800–1,500 ms). The FRN was measured at frontal and frontocentral electrodes (i.e., F3, Fz, F4, FC3, FCz, and FC4). The early and late LPPs were measured at parietal electrodes (i.e., P3, Pz, and P4). The time window for the FRN was chosen on the basis of peaks identified in the grand waveforms across conditions, electrodes of interest, and participants (314 ms); and the time windows for early and late LPP were selected based on the visual inspection of the grand waveforms. Electrodes of interest were selected based on previous studies (e.g., Bellebaum and Daum, 2008; Wu and Zhou, 2009; Walsh and Anderson, 2012; Meadows et al., 2016). Grand-averaged waveforms in gain and loss frames are shown in Figures 3, 4, respectively, and topography maps in Figure 5.

**FIGURE 6:**
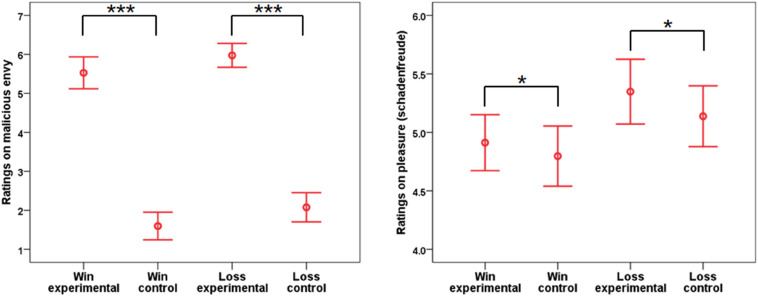
Topographical maps based on the mean amplitude of feedback-related negativity (FRN) and early and late positive potentials (LPPs) for all experimental conditions.

### Data Analysis

**FIGURE 7:**
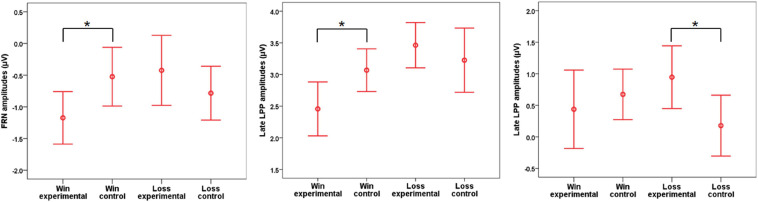
Mean amplitudes of feedback-related negativity (FRN) (the left panel), early late positive potential (LPP) (the middle panel), and late LPP (the right panel) in each experimental condition. Error bars show 95% confidence intervals and are adjusted for within-subject designs. The symbol “*” indicates *p* < 0.05. Note that the significant values refer to the emotional effect in either the gain (FRN and early LPP) or loss (late LPP) frame.

For the behavioral data, ratings on malicious envy and schadenfreude were separately performed by 2 × 2 analyses of variance (ANOVA) with frame (gain versus loss) and emotion (experimental versus control) as the within-subject factors. The *means* and *SE*s of the ratings are shown in Figure 6. With regard to ERPs, we averaged the amplitudes for all electrodes of interest for each ERP component. The averaged amplitudes of FRN and early and late LPPs were separately assessed by using the same ANOVA as that for behavioral data. The means and *SE* of the amplitudes of FRN and early and late LPPs for each condition are shown in Figure 7. Greenhouse–Geisser corrections and Bonferroni corrections were applied to correct degrees of freedom and/or *p* values when appropriate. Statistical analyses were performed, using IBM SPSS Statistics software (Version 22; SPSS Inc., an IBM company, Chicago, IL, United States).

## Results

### Behavioral Data

#### Ratings on Malicious Envy

This analysis aimed to understand whether malicious envy was elicited successfully. The results showed the main effects of emotion [*F*(1,31) = 171.38, *p* < 0.001, η^2^p = 0.85] and frame [*F*(1,31) = 10.49, *p* = 0.003, η^2^p = 0.25]. In general, the ratings were higher in the experimental condition than in the control condition and in the loss frame than in the gain frame. The interaction between emotion and frame was not significant [*F*(1,31) = 0.02, *p* = 0.904, η^2^p < 0.01].

#### Ratings on Pleasure (Schadenfreude)

The results showed that feelings of pleasure (schadenfreude) were stronger in the experimental condition than in the control condition [*F*(1,31) = 4.73, *p* = 0.037, η^2^p = 0.13]. The main effect of frame [*F*(1,31) = 3.01, *p* = 0.093, η^2^p = 0.09] or the interaction between frame and emotion was not significant [*F*(1,31) = 0.26, *p* = 0.612, η^2^p = 0.01].

### ERP Data

#### FRN

The results on FRN responses showed that there was no main effect of frame [*F*(1,31) = 0.67, *p* = 0.420, η^2^p = 0.02] or emotion [*F*(1,31) = 0.29, *p* = 0.592, η^2^p = 0.01] but rather an interaction between those two factors [*F*(1,31) = 5.22, *p* = 0.029, η^2^p = 0.14]. Further analysis showed that, for the gain frame, the FRN was shifted to a more negative direction in the experimental condition than in the control condition [*F*(1,31) = 4.38, *p* = 0.045, η^2^p = 0.12], whereas the emotional effect was not significant in the loss frame [*F*(1,31) = 0.90, *p* = 0.351, η^2^p = 0.03].

#### Early LPP

There was a two-way interaction between emotion and frame [*F*(1,31) = 4.94, *p* = 0.034, η^2^p = 0.14]. Further analysis showed that, for the gain frame, the response of early LPP was more positive in the control condition than in the experimental condition [*F*(1,31) = 6.93, *p* = 0.013, η^2^p = 0.18], whereas the emotional effect was not significant in the loss frame [*F*(1,31) = 0.57, *p* = 0.46, η^2^p = 0.02]. There was no significant effect of frame [*F*(1,31) = 3.86, *p* = 0.059, η^2^p = 0.11] or emotion [*F*(1,31) = 0.90, *p* = 0.351, η^2^p = 0.03].

#### Late LPP

ANOVA showed that there were no main effects of emotion [*F*(1,31) = 0.83, *p* = 0.370, η^2^p = 0.03] or frame [*F*(1,31) < 0.01, *p* = 0.984, η^2^p< 0.01], whereas the interaction between these two factors was significant [*F*(1,31) = 4.31, *p* = 0.046, η^2^p = 0.12]. Further analysis showed that, in the loss frame, the response of late LPP was more positive in the experimental condition than in the neutral condition [*F*(1,31) = 4.61, *p* = 0.040, η^2^p = 0.13], whereas the emotional effect was not significant in the gain frame [*F*(1,31) = 0.35, *p* = 0.558, η^2^p = 0.01].

### A *Post hoc* Power Analysis

In order to understand whether the actual sample and effect sizes can achieve adequate power, we conducted a *post hoc* power analysis by using G^∗^Power 3.1.9.2 software (Faul et al., 2007). The main aim of the present study was to investigate neural effects of malicious envy on schadenfreude dependently on frame. Thus, we used the effect size of this interaction (η^2^p in SPSS = 0.12–0.14) to perform a *post hoc* power analysis. Results showed that the power for the interaction was higher than 0.85, suggesting the actual sample size (i.e., 32) and the acquired effect size in the present study were sufficient.

## Discussion

The present study investigated the modulation of the contextual frame on the behavioral and ERP effects of malicious envy on schadenfreude when malicious envy was elicited in the context of real social comparisons. Behavioral results showed that malicious envy increased feelings of schadenfreude irrespective of the frame. Moreover, ERP results showed that, in the gain frame, ERP responses from FRN to early LPP time ranges were shifted to a more negative direction in the experimental condition than in the control condition when a misfortune occurred to enviable persons, whereas the experimental condition resulted in a larger late LPP response than did the control condition in the loss frame. The findings might suggest that the contextual frame influences the neural effects of malicious envy on schadenfreude.

The effect of malicious envy on feelings of schadenfreude in the present study might be associated with the achievement of the motivation goal of malicious envy. Malicious envy is elicited when others are superior to individuals themselves. Under the influence of malicious envy, individuals have a strong motivation to damage the superior position of others (Van de Ven et al., 2015). A misfortune occurring to others is helpful for the achievement of this goal, thus resulting in increasing pleasure of one (i.e., schadenfreude).

However, behavioral results did not show a modulation of the contextual frame on the effect of malicious envy on schadenfreude. As mentioned in the section “Introduction,” the modulation of the contextual frame might be reflected in two different ways: first, the effect of malicious envy on schadenfreude might be stronger in the gain frame than in the loss frame; and second, the ERP effect in the gain frame might occur at earlier time ranges than the effect in the loss frame. Thus, the absence of the behavioral modulation of the contextual frame in the present study might be because the modulation of the contextual frame is reflected only by the second way. This interpretation can be further validated by ERP results.

The ERP results clearly suggested that the modulation of the contextual frame was reflected by the abovementioned second way, i.e., the ERP effects of malicious envy on schadenfreude occurred at earlier time ranges (i.e., FRN and early LPP) in the gain frame and later time ranges (i.e., late LPP) in the loss frame. Moreover, previous studies have repeatedly suggested that FRN is an indicator of expectancy violation (e.g., Bellebaum and Daum, 2008; Walsh and Anderson, 2012; Meadows et al., 2016). At late time ranges, the LPP is thought to be relevant to outcome evaluations (e.g., Wu and Zhou, 2009; Walsh and Anderson, 2012; Meadows et al., 2016). Nevertheless, early LPP might overlap with the FRN and thus might also be associated with expectancy violation. Taken together, the findings in the present study suggest that, in the gain frame, the misfortune of the player is more likely to violate expectations of the participants when the player previously obtained a superior position (i.e., in the malicious envy condition) than when the player did not (i.e., the control condition); in the loss frame, the participants evaluate misfortune of the player as more positive while experiencing malicious envy.

When the player in the experimental condition obtained a superior position during Phase 1 of the game, the participants might expect that the superior position would be sustained in later Phase 2. However, this expectation is not met when the superior position of the player was damaged by his/her misfortune. In contrast, the participants in the control condition might not expect that the player obtained a superior position in Phase 2 due to his/her misfortune during Phase 1. Thus, the participants did not feel surprised when the player encountered a misfortune once again during Phase 2. The stronger degree of expectancy violation in the experimental condition might thus result in larger neural responses from FRN to early LPP time ranges (in the gain frame).

In addition, the misfortune of the player was beneficial to damaging his or her superior position in the experimental condition. In the control condition, however, the misfortune did not help much to damage the position of the player, as his or her original position had been not superior. Therefore, the misfortune might be evaluated as more positive in the experimental condition than in the control condition and thus elicit stronger responses in the late LPP time range (in the loss frame).

Furthermore, the FRN and (early and late) LPP effects of malicious envy on schadenfreude were distinctive between gain and loss frames. It has been suggested that individuals are more sensitive and pay more attention to outcomes of others in the gain frame than in the loss frame (De Dreu et al., 1992, 1994; Poppe and Valkenberg, 2003). Accordingly, after the player encountered a misfortune in the present study, the participants in the gain frame might pay attention toward the self-irrelevant misfortune immediately after its occurrence (e.g., when salaries increase, one would like to know how much the salary of the superior colleagues increases in order to know the difference in social status between themselves and their colleagues), whereas this might not be the case for the participants in the loss frame (e.g., when salary declines, individuals might not care about social status but rather about whether the decrease in salary impacts their life). The different attentional focuses might result in the effect of malicious envy on schadenfreude, occurring at relatively early time ranges (i.e., FRN and early LPP) in the gain frame and at a late time range (i.e., late LPP) in the loss frame.

In addition, due to attentional focuses on outcomes of others, the participants in the gain frame of the present study might expect what the outcome of the player would be before Phase 2 (e.g., when individuals are told that their salary will increase, they might think that the rates of salary increase will be higher for superior colleagues) and subsequently, compare the expected outcome with the actual outcome after Phase 2. Such expectation and comparison processing might result in the effect of malicious envy on schadenfreude, occurring at expectancy (-violation)-related time ranges in the gain frame. In the loss frame, however, self-focus might allow the participants to concentrate on evaluating how the outcome of the player has an impact on participants themselves after Phase 2 (e.g., salary-declining individuals think that the decline of their salary seems to be not that much, as the salaries of other superior colleagues have also declined to a large extent). Such evaluations might result in the effect of malicious envy on schadenfreude, occurring at outcome-evaluation-related time ranges in the loss frame.

In general, the findings obtained in this study appear to be in line with the theories on envy [e.g., the malicious envy theory (Miceli and Castelfranchi, 2007; Smith and Kim, 2007), the dual envy theory (Van de Ven et al., 2009; Falcon, 2015), and the pain-driven dual envy theory (Lange et al., 2018)], and some empirical studies (e.g., Van Dijk et al., 2006; Takahashi et al., 2009; Cikara and Fiske, 2013; Feather et al., 2013; Van de Ven et al., 2015; Baez et al., 2016, 2018; Santamaría-García et al., 2017), which generally suggest that envy, particularly malicious envy, influences feelings of schadenfreude and neural activity when enviable persons encounter a misfortune. For some empirical studies, however, (malicious) envy was not elicited in the context of real social comparisons, which had reduced ecological validity. By using a real social comparison task, the findings still showed a clear effect, further validating the effect of malicious envy on schadenfreude.

More importantly, the current findings might reveal new insights, demonstrating that the effects – at least the ERP effects – of malicious envy on schadenfreude are influenced by the contextual frame. In the gain frame, the effect of malicious envy on schadenfreude takes place at expectancy-violation-related (i.e., FRN and early LPP) time ranges; the effect occurs at outcome-evaluation-related (e.g., late LPP) time ranges in the loss frame. The findings might extend the theories on envy (e.g., Miceli and Castelfranchi, 2007; Smith and Kim, 2007; Van de Ven et al., 2009; Falcon, 2015; Lange et al., 2018), in which the contextual frame influences the processing stages of the effect of malicious envy on schadenfreude.

Finally, we would like to mention several limitations to the present study and suggest outlines of research in the future studies. First, in addition to envy, schadenfreude is also thought to be a social-comparison-based emotion (Smith, 2000). Little is known about the effect of envy on schadenfreude when schadenfreude is assessed by social comparisons. Future studies might further investigate the related issue. Second, malicious envy and schadenfreude are elicited within the same frame. In the future studies, we might further investigate the effect of malicious envy on schadenfreude when the frames of these emotions are different (e.g., malicious envy was elicited by the player gaining more money than the participants, and schadenfreude by the player losing a large amount of money). Last, for the paradigm, there might be several other approaches that might elicit larger effects of malicious envy on schadenfreude, e.g., enlarge the differences in monetary gain or loss between participants and players (e.g., tell the participants that they receive the general gain or loss across all trials rather than 50% of them), present an inferior outcome for the participants in Phase 1 for more times (i.e., the participants gain less/lose more money than the player several times) and/or a larger misfortune for the player in Phase 2, and increase social competitions between the participants and the players (e.g., if one selects a box, then the other has to select the other box).

## Conclusion

The findings in the present study suggested that malicious envy increased feelings of schadenfreude and ERP responses when a misfortune occurred to enviable persons. More importantly, the contextual frame influenced the processing stages of the effect of malicious envy on schadenfreude. These findings might expand our understanding of the influence of envy on other emotions.

## Data Availability Statement

The raw data supporting the conclusions of this article will be made available by the authors, without undue reservation.

## Ethics Statement

The studies involving human participants were reviewed and approved by the Academic Committee of School of Public Administration, Guangdong University of Finance. The patients/participants provided their written informed consent to participate in this study.

## Author Contributions

HL contributed to the conception and design of the study and wrote the first draft of the manuscript. HL and JL performed the data collection and statistical analysis. Both authors contributed to manuscript revision and read and approved the submitted version.

## Conflict of Interest

The authors declare that the research was conducted in the absence of any commercial or financial relationships that could be construed as a potential conflict of interest.

## Publisher’s Note

All claims expressed in this article are solely those of the authors and do not necessarily represent those of their affiliated organizations, or those of the publisher, the editors and the reviewers. Any product that may be evaluated in this article, or claim that may be made by its manufacturer, is not guaranteed or endorsed by the publisher.
